# Regional-local urinary antibiograms for long-term care homes: a population-wide cross-sectional study

**DOI:** 10.1128/jcm.01223-25

**Published:** 2026-04-27

**Authors:** Bradley J. Langford, Kevin A. Brown, Sarah Swayze, Lucas Castellani, Dan Dalton, Fiona Emdin, Ian Langleben, Samantha Lee, Valerie Leung, Larissa Matukas, Anjali Oberai, Kevin L. Schwartz, Nick Daneman

**Affiliations:** 1 Public Health Ontario 153300, Toronto, Ontario, Canada; 2 Health Research Methods, Evidence, and Impact, McMaster University, Hamilton, Canada; 3 Dalla Lana School of Public Health 153300, Toronto, Ontario, Canada; 4 ICES, Toronto, Canada; 5 Northern Ontario School of Medicine 26627, Greater Sudbury, Ontario, Canada; 6 CareRx Corporation, Toronto, Canada; 7 Institute of Health Policy, Management, and Evaluation 153300, Toronto, Ontario, Canada; 8 Global Strategy Lab 568223 , Toronto, Ontario, Canada; 9 Faculty of Medicine, University of Toronto 7938, Toronto, Ontario, Canada; 10 Toronto East Health Network 27374, Toronto, Ontario, Canada; 11 Department of Laboratory Medicine and Pathobiology, University of Toronto 7938, Toronto, Ontario, Canada; 12 Wawa Family Health Team, Wawa, Canada; 13 Unity Health Toronto, Toronto, Canada; 14 Sunnybrook Health Sciences Centre 71545, Toronto, Ontario, Canada; Vanderbilt University Medical Center, Nashville, Tennessee, USA

**Keywords:** antimicrobial stewardship, long-term care, antibiograms

## Abstract

Antibiograms are challenging to construct in long-term care (LTC) homes in part due to low isolate counts and limited precision. Regional-local antibiograms offer a potential solution by using partial pooling, combining data from both the home and the broader LTC population. We developed regional-local urinary antibiograms and compared this approach to standard antibiograms. This cross-sectional study included urine cultures from LTC residents across Ontario. (i) Standard syndromic combined antibiograms were created for each LTC home with ≥30 total isolates. Homes with <30 isolates used the mean of susceptibility for each drug for the entire province. (ii) Partially pooled regional-local antibiograms were constructed using logistic mixed models with a random intercept for each home to ensure each LTC home could be provided a facility-specific antibiogram. We compared susceptibility and rank order of recommended antibiotics using each method. Among 627 LTC homes, 340 (54.2%) met the ≥30 isolate threshold. Regional-local methods allowed for the development of 627 LTC home-specific antibiograms. These methods narrowed susceptibility estimate ranges compared to standard methods (e.g., trimethoprim-sulfamethoxazole [TMP-SMX]: 57%–77% vs 37%–90%, respectively). A total of 119 (19.0%) homes had at least one antibiotic with over 80% susceptibility using the standard approach; only 11 (1.8%) homes met this threshold with the regional-local antibiogram. The antibiotic with the highest susceptibility varied based on methodology (amoxicillin-clavulanate for standard vs TMP-SMX for regional-local antibiogram). Regional-local antibiograms allow for the creation of facility-specific antibiograms, even among facilities with small isolate counts. This approach may provide more precise antibiotic susceptibility estimates by reducing misleading inter-facility variation.

**IMPORTANCE:**

Local antibiograms can help inform empiric antibiotic treatment for long-term care residents with urinary tract infections. However, many facilities have too few isolates to create precise susceptibility estimates. A regional-local antibiogram is a novel strategy that uses a weighted approach: it prioritizes local data where available, but increasingly uses regional population-level data for homes with smaller sample sizes to improve statistical precision and reduce misleading inter-facility variation. This regional-local approach allows for the development of facility-specific antibiograms for a greater number of homes compared to traditional methods of developing antibiograms.

## INTRODUCTION

Cumulative antibiograms help institutions and regions track antimicrobial resistance (AMR) to better understand epidemiological trends and support optimal empiric prescribing of antimicrobials (1). Most local and regional antibiograms focus on hospitals and/or the community, excluding long-term care (LTC) settings in which older residents, congregate living environments, and high levels of inappropriate antimicrobial use may lead to a high prevalence of AMR (2).

A challenge in developing facility-specific antibiograms is that LTC homes may have an insufficient number of isolates necessary to inform a meaningful estimate of antimicrobial susceptibility (3). Clinical and Laboratory Standards Institute (CLSI) publishes M39 Analysis and Presentation of Cumulative Antimicrobial Susceptibility Test Data to provide guidance for facilities developing antibiograms (4). A key recommendation is to only include species with 30 or more isolates during a given analysis period to ensure a precise statistical estimate of susceptibility. Since many LTC homes will have fewer than 30 positive isolates for each species annually, several approaches have been suggested to provide a more adequate sample size, including using data from local referral hospitals, older patients residing in the community, and extending the period of observation to more than 1 year (3, 4). However, these approaches may over- or underestimate the true rates of AMR and mask rapidly evolving trends (5).

Another strategy to overcome low isolate counts is the use of a weighted incidence syndromic combined antibiogram (WISCA) (3, 6). Compared to standard antibiogram tables, in which susceptibility is provided for each organism-drug combination, a WISCA provides susceptibility for all organisms combined, weighted by their incidence, for each drug for a given syndrome (e.g., urine cultures). Using a WISCA provides a larger overall sample size, offering a more accurate and more clinically meaningful estimate of susceptibility compared to focusing on a single organism. This may ease the cognitive burden on prescribers by providing a single point estimate of overall susceptibility to a particular antibiotic rather than multiple rows of organism-specific data (e.g., overall 80% of urinary isolates are susceptible to nitrofurantoin in a WISCA vs 95% of *Escherichia coli*, 50% of *Klebsiella* spp., and 0% of *Pseudomonas aeruginosa* are susceptible to nitrofurantoin in a standard antibiogram). However, in smaller facilities or those with a lower rate of urine culturing, the total count may still be fewer than 30 even when combining all organisms.

One solution is to use regional-local antibiograms, which leverage partial pooling by combining data from both a specific LTC home and the broader LTC population. Regional-local antibiograms may help to increase the precision of susceptibility estimates while still providing useful data specific to the unique ecology of each home. Such approaches could provide more LTC homes with tailored data to increase the number of organism-drug combinations meeting the CLSI-recommended threshold. However, to our knowledge, such methodologies have yet to be evaluated and compared with standard antibiogram methods.

Given the lack of local capacity to prepare antibiograms and low sample size of isolates in many LTC homes, we undertook an analysis with two main objectives. We aimed to (i) centrally develop regional-local urinary antibiograms for all LTCs across a region—regardless of isolate count—and (ii) compare this approach with the standard CLSI methodology that relies on a threshold of 30 isolates for reporting.

## MATERIALS AND METHODS

### Study design and setting

We performed a cross-sectional study of urinary antimicrobial susceptibility data from long-term care home residents from 1 January to 31 December 2021, in Ontario, Canada’s most populous province.

### Participants and eligible cultures

Residents of any Ontario LTC with a positive urine culture and susceptibility data were eligible for inclusion. We excluded urine cultures with possible contaminants (i.e., coagulase-negative Staphylococcus, *Corynebacterium* spp., Streptococcus viridans group, *Actinobaculum* spp., *Aerococcus* spp., yeast/*Candida* spp., and *Lactobacillus* spp.) or mixed growth. We excluded cultures collected outside of the LTC setting unless they were among LTC residents during the first 48 h of hospital visit to capture organisms acquired in long-term care. For patients with multiple occurrences of the same organism during the study period, we selected only the first culture.

### Source of data

Urine culture data were acquired from the Ontario Laboratories Information System (OLIS). OLIS is a province-wide repository for laboratory results across public health and health care sectors. Culture and susceptibility data from more than 95% of clinical laboratories in the province were included (7). We collected reported susceptibility data for the following antibiotics typically used to treat urinary tract infections in LTC residents: amoxicillin; amoxicillin-clavulanate; cephalexin (based on cefazolin or cephalexin result); cefixime; trimethoprim-sulfamethoxazole (TMP-SMX); ciprofloxacin; and nitrofurantoin.

LTC home resident data (including functional status and device dependence) were obtained from the Continuing Care Reporting System LTC and the Registered Persons Database. LTC home location was based on the definition for Ontario Health Regions (8). Comorbidity data were collected from the Canadian Institute for Health Information Discharge Abstract Database. These data sets were linked by unique encoded identifiers and analyzed at ICES. ICES is an independent nonprofit research institute whose legal status under Ontario’s health information privacy law allows it to collect and analyze health care and demographic data, without consent, for the purpose of evaluating and improving the health system.

### Reducing the missingness of data

In OLIS, some susceptibility data may be incomplete due to (i) differential antibiotic susceptibility testing and reporting practices between laboratories and (ii) susceptibility data being based on reported results, rather than tested results (due to selective/cascade reporting). Therefore, we used a two-step imputation method to reduce missingness. The first step was rule-based imputation based on known intrinsic susceptibility, intrinsic resistance, and cross-susceptibility and resistance (e.g., *E. coli* resistant to amoxicillin-clavulanate will also be resistant to amoxicillin). The second step was model-based imputation to predict susceptibility for organisms with missing data after rule-based imputation. This approach used logistic regression models, including the variables age, sex, region, organism, and available antibiotic test results for other reported agents, to predict antibiotic susceptibility of missing antibiotic agents. The imputation methodology has been previously described in more detail elsewhere (9).

### Analysis

We provide descriptive data on residents and LTC homes included in the analysis.

### Standard antibiogram methodology

A combined urinary antibiogram (WISCA) was calculated for each LTC home. This combined urinary antibiogram combined the susceptibility estimates for all organisms into a single row for each drug for each home. The combined susceptibility was calculated based on the percentage of organisms susceptible to a given drug weighted by the organism’s incidence. In alignment with CLSI M39 recommendations, the facility-specific combined antibiogram was only reported for homes in which the total number of urinary isolates was at least 30 (4). In situations where the facility-specific total count was less than 30, mean susceptibility data for each organism-drug combination for the entire province were used. For example, if a home had only 18 isolates, we did not report the facility-specific WISCA percent susceptibility, but rather the grand mean percent susceptibility WISCA for the province (however, these 18 isolates contribute to the grand provincial mean).

### Regional-local antibiogram

In addition to the standard approach described above, we constructed a partially pooled model that uses susceptibility information from both the local LTC home and from LTC residents across the entire province (the grand mean of susceptibility for each organism-drug combination). This was implemented as a series of logistic mixed models where, for each organism-drug combination, the outcome was the count of resistant and susceptible isolates, with an intercept representing the grand mean, and random intercepts for each LTC home. These models pool susceptibility estimates for each organism-drug towards the grand mean, where there is greater pooling for homes with smaller counts of urinary isolates and less for homes with higher counts of urinary isolates. This method was performed for all homes, regardless of isolate count (see Table 1 for comparison of methodology).

**TABLE 1:** Comparing regional-local to standard antibiogram methodologies^*a*^

Characteristic	Standard antibiogram	Regional-local antibiogram
Data source	Specific LTC home OR LTCs in province (if sample size is <30)	Specific LTC home AND LTCs in province
Data reporting threshold	Only reports facility-specific data if ≥30 isolates; otherwise uses provincial average	Incorporates data from all homes regardless of isolate count
Methodology	WISCA with home-specific data	WISCA with home and province-wide data; logistic regression model which partially pools data from LTC and province (greater pooling for homes with smaller counts of urinary isolates)

^*a*^ LTC, long-term care; WISCA, weighted incidence syndromic combination antibiogram.

### Comparing antibiogram strategies

To compare the regional-local antibiogram to the standard approach, we determined the number of LTC institutions with 30 or more isolates in the combined antibiogram (i.e., sufficient data to provide a facility-specific antibiogram). To compare the central tendency and range of susceptibility for each drug, we constructed box plots for antibiogram susceptibility using both standard and regional-local methodologies. To determine if there was a difference in first-line recommended antibiotics based on methodology, we ranked each antibiotic based on susceptibility (highest to lowest) for each home and compared the antibiotics with the highest susceptibility between the two methodologies. We also determined the number of antibiotics with susceptibility ≥80% according to each antibiogram method, given that this is a common threshold used to identify potentially active empiric urinary tract infection treatment options (4, 5).

## RESULTS

**Fig 1:**
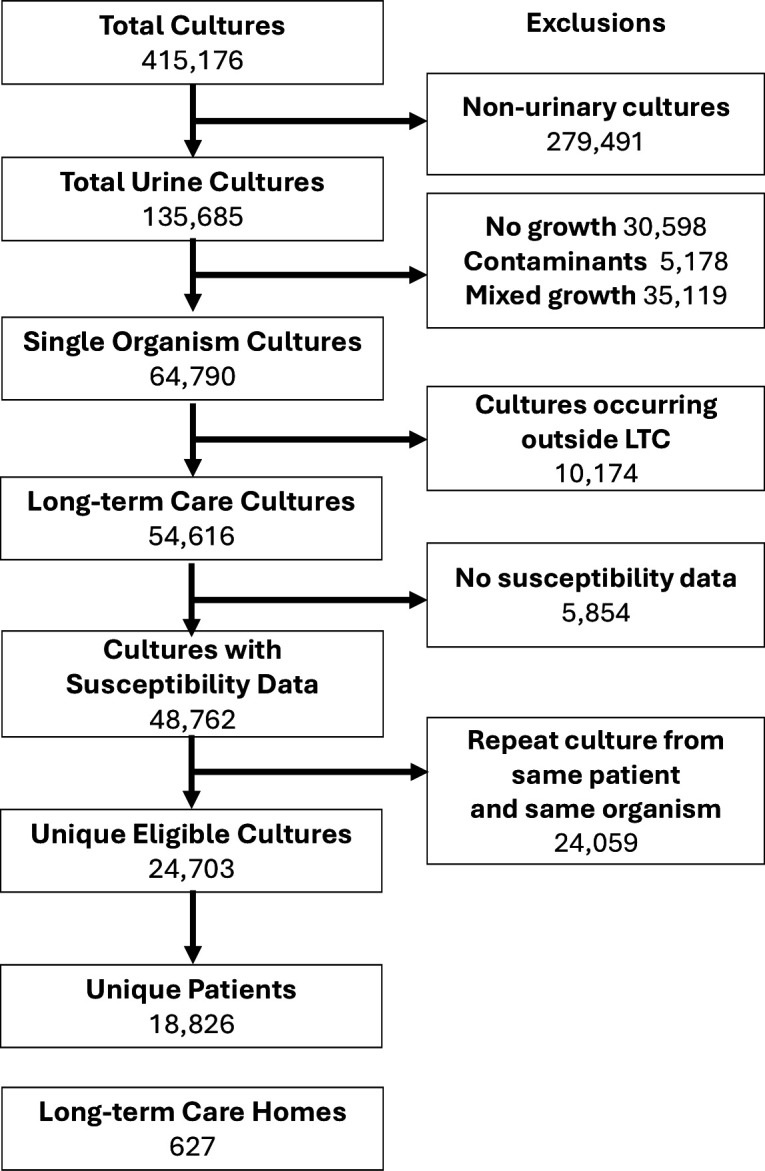
Study flow diagram. LTC, long-term care.

We included 24,703 unique positive urine cultures from 18,826 residents residing in 627 long-term care homes (Fig. 1). Residents were mostly female (median 76%), and the median age was 84 years. Bladder incontinence was also very common (median 90%). Urinary catheter use was relatively uncommon (median 13%). Hospital visits in the last 90 days were infrequent (median 6%; more details provided in Table 2).

**TABLE 2:** Characteristics of included long-term care homes^*a*^

Characteristic		Regional-local antibiograms *N* = 627	Standard antibiograms *N* = 340
Region	West	237 (37.8%)	114 (33.5%)
	Central	101 (16.1%)	68 (20.0%)
	Toronto	83 (13.2%)	58 (17.1%)
	East	148 (23.6%)	78 (22.9%)
	North East	43 (6.9%)	17 (5.0%)
	North West	15 (2.4%)	≤5 (1.5%)
Bed size	Large (≥100)	377 (60.1%)	288 (84.7%)
	Medium (30–99)	233 (37.2%)	52 (15.3%)
	Small <30	17 (2.7%)	0 (0.0%)
Number of cultures	<30	287 (45.8%)	0 (0.0%)
	30–50	177 (28.2%)	177 (52.1%)
	51–100	131 (20.9%)	131 (38.5%)
	>101	32 (5.1%)	32 (9.4%)
Proportion female	Mean ± SD	75.67 ± 11.71	75.41 ± 9.11
	Median (IQR)	76 (68–82)	76 (70–81)
Mean age	Mean ± SD	83.59 ± 3.74	84.05 ± 2.69
	Median (IQR)	84 (82–86)	84 (83–86)
Mean Charlson Index	Mean ± SD	1.85 ± 0.64	1.86 ± 0.43
	Median (IQR)	2 (1–2)	2 (2–2)
Bowel incontinence (percentage of patients)	Mean ± SD	74.91 ± 14.09	76.84 ± 10.99
	Median (IQR)	77 (67–85)	78 (70–85)
Bladder incontinence (percentage of patients)	Mean ± SD	88.04 ± 10.11	88.55 ± 8.08
	Median (IQR)	90 (83–95)	90 (84–94)
Hospital visit in last 90 days (percentage of patients)	Mean ± SD	8.83 ± 10.56	5.65 ± 4.84
	Median (IQR)	6 (3–11)	4 (3–7)
Indwelling catheter (percentage of patients)	Mean ± SD	13.70 ± 8.75	12.13 ± 6.45
	Median (IQR)	13 (8–17)	12 (7–16)

^*a*^ SD, standard deviation; IQR, inter-quartile range.

Of the 24,703 positive cultures, the most commonly isolated organisms were *E. coli* (11,997, 48.6%), *Klebsiella pneumoniae* (3,346, 13.5%), and *Proteus mirabilis* (2,890, 12.1%). Overall grand-mean susceptibility across all organisms in the combined antibiogram (WISCA) was highest to TMP-SMX and amoxicillin-clavulanate, both at 71%, followed by nitrofurantoin and first-generation cephalosporins, both at 68%.

### Comparing regional-local vs standard antibiograms

#### Number of LTC homes with sufficient data to create an antibiogram by each method

The number of positive urine cultures varied across homes from <6 to 290. Median positive urine culture count was 33, and the interquartile range was 18–51. Using the CLSI-recommended threshold of 30 total isolates or more, 340 (54%) LTC homes had an adequate sample of isolates to develop and report a facility-specific combined antibiogram. Thus, the remaining 287 homes were assigned the grand mean of susceptibility for the province for their standard antibiogram.

#### Overall antibiotic susceptibilities by each method

**Fig 2:**
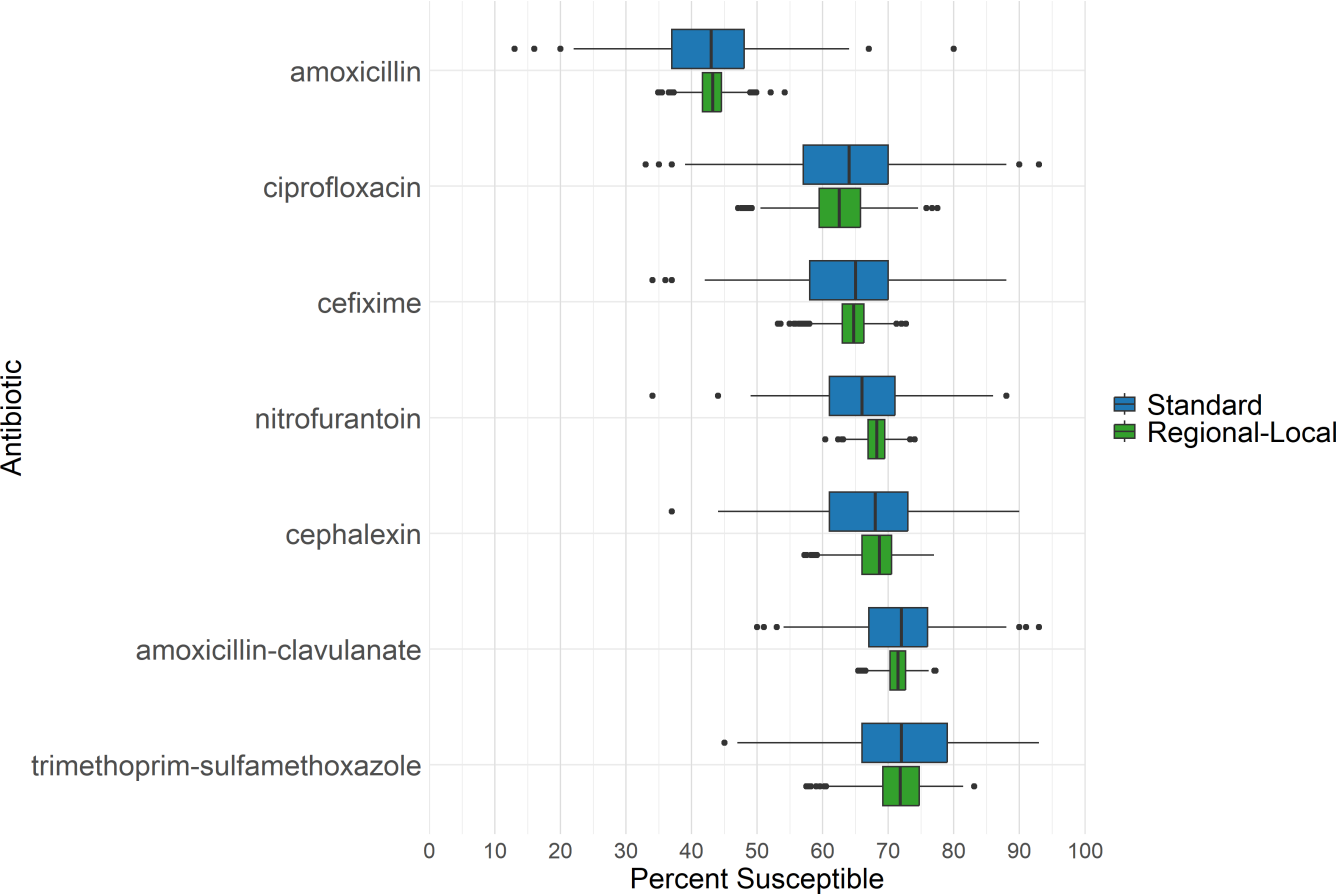
Combined urinary antibiotic susceptibility across long-term care homes using different antibiogram methodologies. Boxplots illustrate the percent susceptibility to all included urinary organisms for each antibiotic. Plots display susceptibility across homes by median (vertical line in the middle of the box), first and third quartiles (left and right ends of the box), minimum and maximum excluding outliers (left and right ends of the horizontal line), and outliers (those beyond 1.5× the interquartile range) represented by dots to the left and right of the horizontal line.

While the median antibiotic susceptibility remained similar across all drugs using either method, the ranges were narrower with the use of the regional-local antibiogram. For example, the ranges for TMP-SMX were 37%–90% susceptible using the standard approach and 57%–77% susceptible using the regional-local antibiogram. Similarly, for amoxicillin-clavulanate, the range in susceptibility was 50%–93% using the standard approach and 65%–77% using the regional-local antibiogram (Table S2 describes percent susceptibility variability for all LTC homes [2a], those with at least 30 isolates [2b], and those with fewer than 30 isolates [2c]; Fig. 2).

#### Number of antibiotics exceeding 80% threshold by each method

While 119 (19.0%) homes had at least one antibiotic over the susceptibility threshold of 80% using the standard approach, only 11 (1.8%) homes met this threshold with the regional-local antibiogram.

#### Highest susceptibility antibiotics by each method

**Fig 3:**
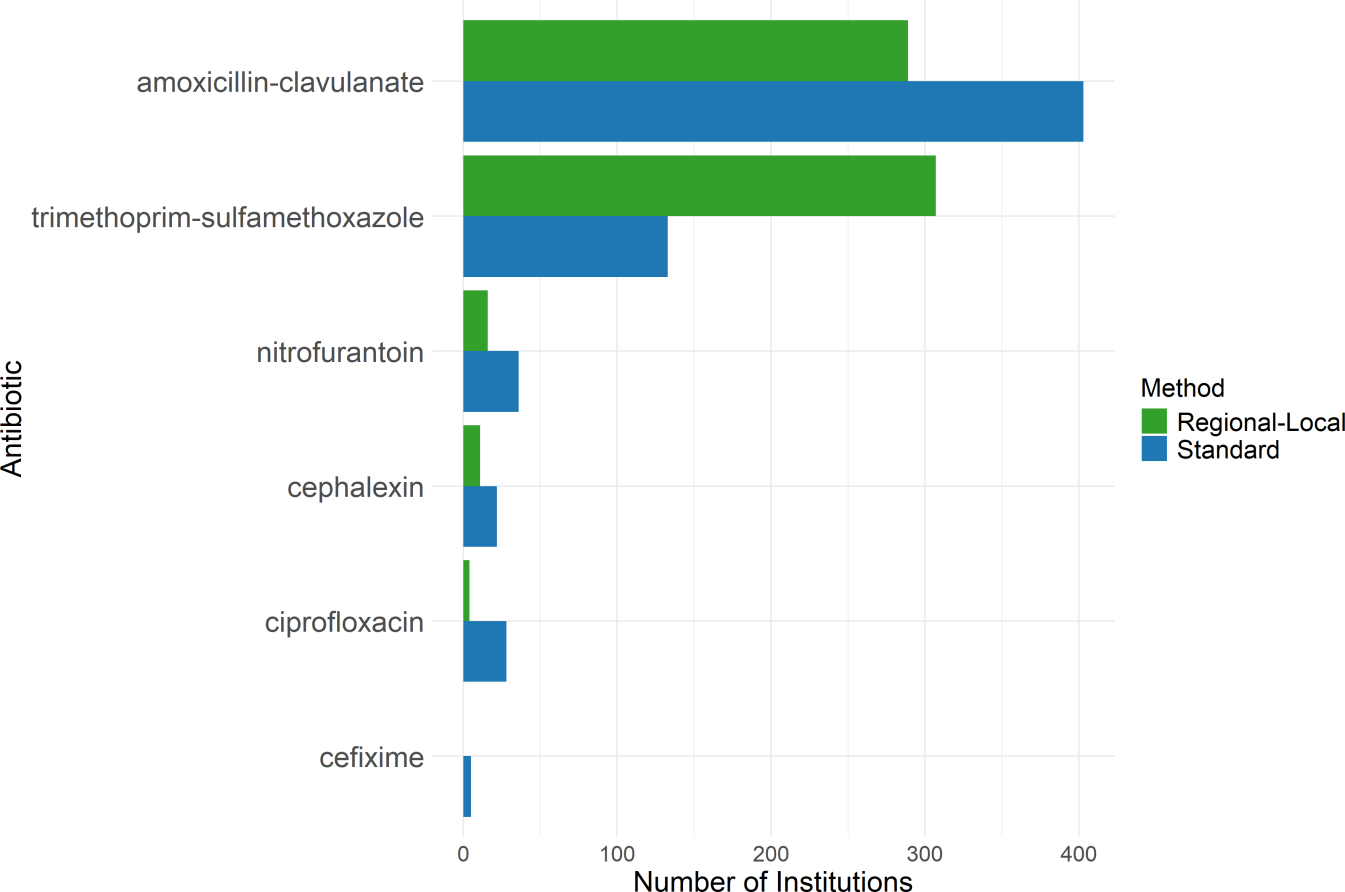
Antibiotics with the highest susceptibility across long-term care homes using different antibiogram methodologies. A bar graph indicates the antibiotics with the highest rank susceptibility for all urinary organisms (WISCA) across institutions. Larger bars indicate more institutions report this antibiotic as the highest-ranked susceptibility across all antibiotics listed.

When ranking antibiotics from highest to lowest susceptibility, the antibiotic with the highest susceptibility varied based on methodology. Highest susceptibility for standard antibiograms included amoxicillin-clavulanate (403 homes, 64.2%), TMP-SMX (133 homes, 21.2%), nitrofurantoin (36 homes, 5.7%), and ciprofloxacin (28 homes, 4.4%). The highest susceptibility for the regional-local methodology included TMP-SMX (307 homes, 49.0%), amoxicillin-clavulanate (289 homes, 46.1%), nitrofurantoin (16 homes, 2.6%), and first-generation cephalosporins (11, 1.8%; Fig. 3). The highest-ranked antibiotic between the two methods was the same (e.g., TMP-SMX for standard and TMP-SMX for regional-local) in 383 homes (61.1%). Among the 244 (38.9%) homes with different first-ranked antibiotics, the most common difference was amoxicillin-clavulanate being top ranked for standard and TMP-SMX being top ranked for regional-local (*n* = 151, 24.1%).

Antibiotics that were second highest in susceptibility were numerically close to those with the highest susceptibility. The difference between first- and second-highest antibiotics by susceptibility was less than 5% for 475 homes (75.8%) using the standard method and for 522 homes (83.3%) using the regional-local method. Figure S1 shows the frequency of first- and second-ranked antibiotics.

## DISCUSSION

This cross-sectional study found that a regional-local syndromic antibiogram using partial pooling provided all homes, regardless of their count of urinary isolates, with a facility-specific antibiogram informed by both local and regional susceptibility data. Almost half of all LTC homes had fewer than 30 urinary isolates, which, when using standard approaches, compromises the precision of the susceptibility estimates and may lead to spurious variability resulting in over- or under-estimates in antibiotic susceptibility for each LTC home (10). Over-reliance on facility-level antibiograms may give a false impression of wide inter-facility variability due to small sample sizes and imprecise estimates. This wide variability in susceptibility was evident even amongst homes with over 30 isolates, where low numbers of isolates may still lead to distorted susceptibility estimates. As such, strategies to supplement data, such as partial pooling, are needed to provide institutions with meaningful estimates of antibiotic susceptibility.

In LTC homes, the development of local antibiograms is challenging for several reasons. Most long-term care homes lack on-site microbiology laboratories, which limits the collaboration and communication necessary to develop, disseminate, and provide education on the use of these tools (11). Furthermore, infectious diseases expertise needed to interpret and translate antibiogram data into actionable recommendations is typically lacking in these settings. Providing facility-specific antibiograms to LTC homes can help to improve decision-making and selection of active, narrower-spectrum empiric therapy (12). However, with a small sample size of isolates, standard antibiograms are often not feasible. Regional-local antibiograms can be facilitated by centralized development, while incorporating enhanced features such as combined antibiograms (i.e., WISCA) to support syndrome-specific decision-making. These combined antibiograms may provide a more all-encompassing estimate of susceptibility, accounting for all organisms. Focusing exclusively on specific common organisms like *E. coli* may result in inaccurate estimates of the activity of empiric antibiotic therapy, especially in older adults in LTC homes, in which non-*E. coli* pathogens are relatively common (9).

This is one of the largest studies on antibiogram development, benefiting from province-wide administrative laboratory data and linked data sets to precisely identify LTC residents and patient characteristics. However, there are key limitations to this study. There may be variability in susceptibility testing procedures across the many laboratories included in our study, which could include the implementation of interpretive criteria and reporting or suppression of results. However, laboratories in our region generally use CLSI as a standard, and we employed rule and model-based imputation to address missingness and reduce variability between labs. As with any antibiogram, data on patient symptoms are not included. Hence, many positive cultures may represent asymptomatic bacteriuria rather than active infection, which is particularly important in this setting, as asymptomatic bacterial colonization of the urinary tract and subsequent unnecessary antibiotic treatment is particularly common in this population (13). Hence, the interpretation of antibiogram data should account for these limitations, and messaging associated with facility-specific antibiograms should encourage best practices such as ensuring appropriate urine culturing and treatment only among those with local or systemic symptoms of UTI and interpreting antibiogram and culture results in the clinical context of each specific patient.

Another limitation is the lack of outcomes data on the clinical impact of these different approaches to antibiogram development. Given the relatively small differences in susceptibility between many antibiotics (TMP-SMX, amoxicillin-clavulanate, nitrofurantoin, and first-generation cephalosporins, whose median susceptibility all fell within the range of 68%–71%), there may be limited clinical impact of selecting one agent over another. Hence, selection of the appropriate empiric antibiotic should also be guided by patient factors (e.g., allergies, contraindications, previous culture, and antibiotic use) and potential harms (e.g., risk of adverse effects, *C. difficile* infection, and selection for antimicrobial-resistant organisms).

These regional-local antibiograms will be used to provide locally informed recommendations for first-line antibiotic therapy in LTC patients with symptomatic cystitis. We will aim to evaluate the impact of providing these tailored data on prescribing practices.

Future work should focus on patient-specific empiric recommendations, taking into account the range of factors that may impact effectiveness and safety. Research is needed to understand the predictive value of these regional-local antibiograms in comparison to standard approaches (i.e., whether they predict future susceptibility results at the individual and population level) and evaluate the prescribing and clinical impact of these antibiogram enhancements.

### Conclusion

Regional-local antibiograms that partially pool data from both the individual facility and the broader population can help provide facility-specific antibiograms for all long-term care homes, even with total isolate counts below 30. This approach can help increase the precision of susceptibility estimates and minimize spurious variability between homes, which could lead to more contextually relevant and appropriate local recommendations for empiric therapy. Future efforts should evaluate the predictive value and impact of such regional-local antibiograms.

## SUPPLEMENTAL MATERIAL

Supplemental tables and figure jcm.01223-25-s0001.docx Tables S1 and S2, and Figure S1.

ASM does not own the copyrights to Supplemental Material that may be linked to, or accessed through, an article. The authors have granted ASM a non-exclusive, world-wide license to publish the Supplemental Material files. Please contact the corresponding author directly for reuse.
