# Supplementary material for: Regional-local urinary antibiograms for long-term care homes: a population-wide cross-sectional study
Source: J Clin Microbiol. 2026 Apr 27;64(6):e01223-25. doi: 10.1128/jcm.01223-25 (PMC13251378; doi:10.1128/jcm.01223-25)
Supplement: Supplemental tables and figure — Tables S1 and S2, and Figure S1. [file jcm.01223-25-s0001.docx]

**Supplementary Data**

**Table 1. Organism-Specific Counts and Percent Susceptibility among LTC Urinary Isolates**

| ***Organism*** | **Count** |
| --- | --- |
| *Escherichia coli* | 11997 |
| *Klebsiella pneumoniae* | 3346 |
| *Proteus mirabilis* | 2890 |
| *Pseudomonas aeruginosa* | 1196 |
| *Streptococcus group B* | 939 |
| *Enterococcus faecalis* | 766 |
| *Klebsiella oxytoca* | 491 |
| *Enterobacter cloacae* | 453 |
| *Staphylococcus aureus* | 420 |
| *Enterococcus sp. Other* | 372 |
| *Morganella sp.* | 357 |
| *Citrobacter freundii* | 351 |
| *Providencia species* | 314 |
| *Citrobacter koseri* | 200 |
| *Enterobacter aerogenes* | 141 |
| *Enterococcus faecium* | 103 |
| *Citrobacter sp. Other* | 73 |
| *Raoutella species* | 54 |
| *Serratia marcescens* | 46 |
| *Proteus vulgaris* | 40 |
| *Klebsiella sp. Other* | 32 |
| *Streptococcus Group C/D* | 31 |
| *Acinetobacter baumannii complex* | 20 |
| *Enterobacter sp. Other* | 17 |
| *Proteus sp. Other* | 12 |
| *Acinetobacter sp. Other* | 10 |
| *Pantoea species* | 10 |
| *Pseudomonas species* | 7 |
| *Achromobacter species* | <6 |
| *Salmonella enteric sp. non-typhi/paratyphi* | <6 |
| *Serratia sp.* | <6 |
| *Staphylococcus lugdunensis* | <6 |
| *Stenotrophomonas maltophilia* | <6 |

Note: due to privacy policies, cells smaller than 6 were suppressed

**Table 2.**

**2a. Antibiotic Susceptibility Central Tendency for Antibiograms across all LTCs**

|  | Median | | IQR | | Range | |
| --- | --- | --- | --- | --- | --- | --- |
|  | Standard | Regional-local | Standard | Regional-local | Standard | Regional-local |
| amoxicillin | 43 | 43 | 37-48 | 42-45 | 13-80 | 35-54 |
| amoxicillin-clav | 72 | 71 | 67-76 | 70-73 | 50-93 | 65-77 |
| 1GC cephalosporin | 68 | 69 | 61-73 | 66-71 | 37-90 | 57-77 |
| cefixime | 65 | 65 | 58-70 | 63-66 | 34-88 | 53-73 |
| ciprofloxacin | 64 | 63 | 57-70 | 59-66 | 33-93 | 47-78 |
| nitrofurantoin | 66 | 68 | 61-71 | 67-69 | 34-88 | 60-74 |
| TMP-SMX | 72 | 72 | 61-73 | 69-75 | 37-90 | 57-77 |

**2b. LTCs with at Least 30 Isolates: Antibiotic Susceptibility Central Tendency**

|  | Median | | IQR | | Range | |
| --- | --- | --- | --- | --- | --- | --- |
|  | Standard | Regional-local | Standard | Regional-local | Standard | Regional-local |
| amoxicillin | 43 | 43 | 37-48 | 41-45 | 13-80 | 35-54 |
| amoxicillin-clav | 72 | 72 | 67-76 | 70-73 | 50-93 | 65-77 |
| 1GC cephalosporin | 68 | 68 | 61-73 | 65-71 | 37-90 | 57-77 |
| cefixime | 65 | 64 | 58-70 | 62-67 | 34-88 | 53-73 |
| ciprofloxacin | 64 | 63 | 57-70 | 59-67 | 33-93 | 47-78 |
| nitrofurantoin | 66 | 68 | 61-71 | 65-71 | 34-88 | 57-77 |
| TMP-SMX | 72 | 72 | 66-79 | 69-75 | 45-93 | 57-83 |

**2c. LTCs with Fewer than 30 Isolates: Antibiotic Susceptibility Central Tendency**

|  | Median | | IQR | | Range | |
| --- | --- | --- | --- | --- | --- | --- |
|  | Standard | Regional-local | Standard | Regional-local | Standard | Regional-local |
| amoxicillin | 43 | 43 | 43-43* | 42-44 | 43-43* | 38-49 |
| amoxicillin-clav | 71 | 71 | 71-71* | 70-73 | 71-71* | 67-75 |
| 1GC cephalosporin | 68 | 69 | 68-68* | 67-70 | 68-68* | 59-75 |
| cefixime | 64 | 65 | 64-64* | 64-66 | 64-64* | 59-70 |
| ciprofloxacin | 62 | 62 | 62-62* | 60-65 | 62-62* | 48-71 |
| nitrofurantoin | 68 | 68 | 68-68* | 67-69 | 68-68* | 64-72 |
| TMP-SMX | 71 | 72 | 71-71* | 70-74 | 71-71* | 61-81 |

**LTCs with fewer than 30 total isolates were replaced with the grand mean for the standard method*

clav = clavulanate, TMP-SMX= trimethoprim-sulfamethoxazole, 1GC = first generation cephalosporins

**Supplementary Figure 1. Highest and Second-Highest Susceptibility Antibiotics by Methodology***


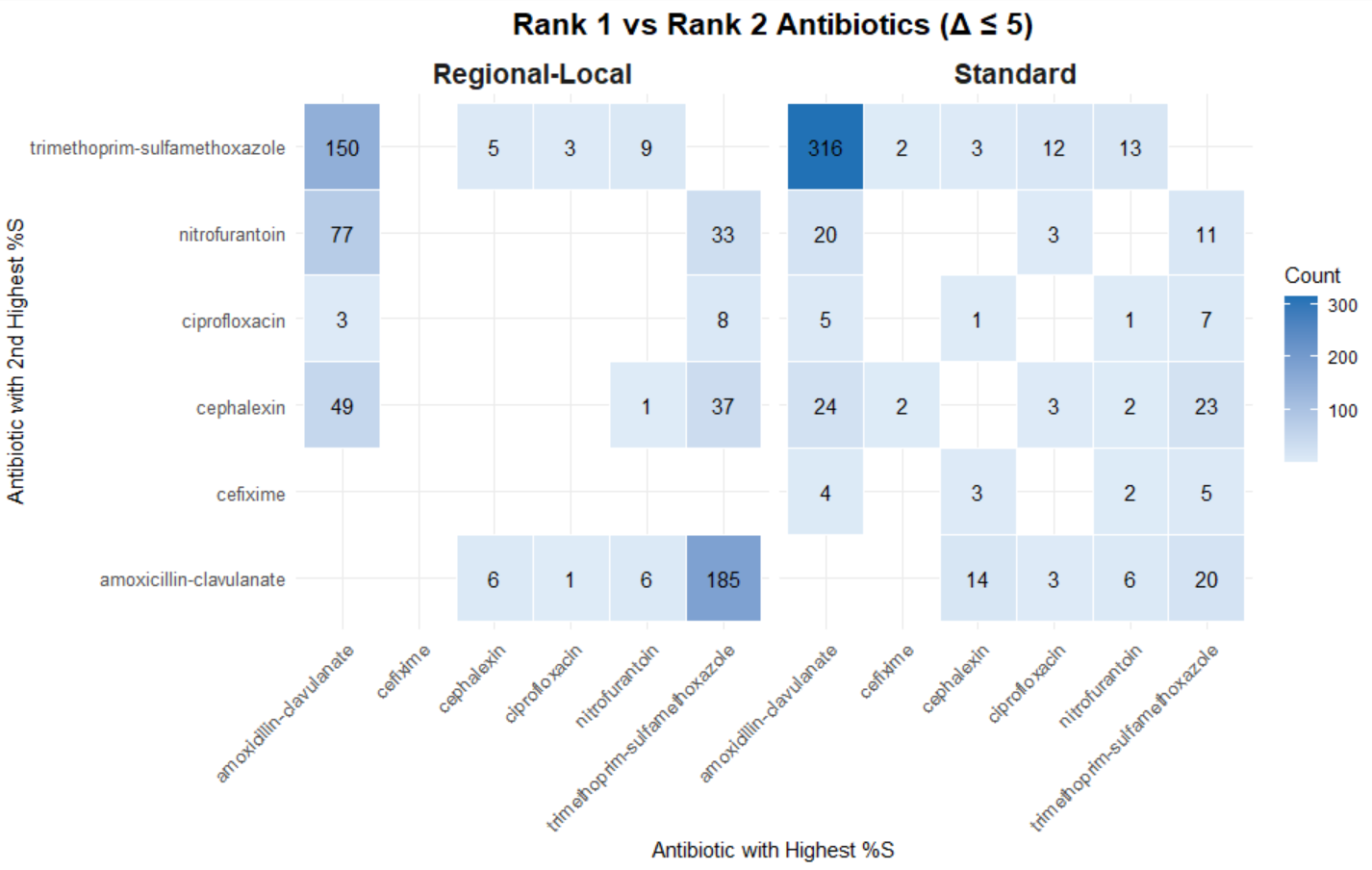


*This figure shows how the two methodologies differ in terms of the highest and second highest ranked antibiotics by percent susceptibility. For example, in the regional-local antibiogram, there are 160 homes with amoxicillin-clavulanate as the highest susceptibility and trimethoprim sulfamethoxazole as the second-highest susceptibility. On the other hand, using the standard method, 318 homes have this same ranking order (amoxicillin-clavulanate first and trimethoprim-sulfamethoxazole second). The figure aims to show how the method selected may impact the choice of empiric agent in each home. Including the second ranked antibiotic may help to decide if the second ranked antibiotic may be more appropriate overall (i.e., whether a small compromise in % susceptibility may be worth reduced risk of side effects or narrower spectrum). *Note this figure includes only LTCH antibiograms in which the difference between highest and second highest is less than or equal to 5%, since homes with a difference >5%, the antibiotic with the highest percent susceptibility may be a more definitive choice for optimal empiric therapy.*
